# How to follow-up a patient who received tocilizumab in severe COVID-19: a case report

**DOI:** 10.1186/s40001-020-00438-x

**Published:** 2020-08-27

**Authors:** Regina B. Podlasin, Justyna D. Kowalska, Andrzej Pihowicz, Beata Wojtycha-Kwaśnica, Magdalena Thompson, Tomasz Dyda, Hanna Czeszko-Paprocka, Andrzej Horban

**Affiliations:** 1 IV-th Department, Hospital for Infectious Diseases, Warsaw, Poland; 2 Department of Adults’ Infectious Diseases, Medical University of Warsaw, Warsaw, Poland; 3 Intensive Care Unit, Hospital for Infectious Diseases in Warsaw, Warsaw, Poland; 4 Department of Diagnostic Imaging, Hospital for Infectious Diseases in Warsaw, Warsaw, Poland; 5 Molecular Diagnostics Laboratory, Hospital for Infectious Diseases in Warsaw, Warsaw, Poland; 6 Central Analytical Laboratory, Hospital for Infectious Diseases in Warsaw, Warsaw, Poland

**Keywords:** SARS-CoV-2, COVID-19, Tocilizumab, Chloroquine, ARDS, Cytokine storm

## Abstract

**Background:**

COVID-19 is characterized by fast deterioration in the mechanism of cytokine storm. Therefore, treatment with immunomodulating agents should be initiated as soon as hyperinflammation is established. Evidence for the use of tocilizumab (TCZ) in COVID-19 is emerging, but the drug in this setting is used “off label” with limited data on both effectiveness and safety. Therefore, Hospital for Infectious Diseases in Warsaw established a Standard Operating Procedure (SOP) for the use of TCZ in severe COVID-19 cases.

**Case presentation:**

Here, we present a case of 27-year-old, otherwise healthy man, who was successfully treated with chloroquine, azithromycin, tocilizumab and a standard of care. Initially the magnitude of lung devastation, clinical deterioration and the need for mechanical ventilation suggested unfavorable prognosis. However, we observed complete regression in radiological changes and rapid clinical improvement. Irrespective of this, patient’s serum interleukin 6 and aminotransferases remained elevated even after a month from treatment.

**Conclusions:**

An overlapping effect of hyperinflammation, hypoxic organ injury and drug-related toxicity warrants a long-term follow-up for COVID-19 survivors. In addition, residual IL-6 receptors blockage may mask new infections. A standardized approach to follow-up for COVID-19 survivors is urgently needed. Current and future research should also investigate the impact of experimental therapies on lung tissue healing and regeneration, as well as long-term treatment toxicities.

## Background

In the course of SARS-CoV-2, most infections are mild or asymptomatic, but up to 25% of hospitalized patients would experience severe complications and progress to critical condition characterized by acute respiratory distress syndrome (ARDS), requiring mechanical ventilation [1]. In the course of Coronavirus Disease 2019 (COVID-19) pathogenic T cells and inflammatory monocytes may enter the pulmonary circulation and initiate the inflammatory storm leading to lung injury and immune disorder in severe COVID-19 patients [2]. Zhang et al. discussed that the sIL-6R (soluble receptor) and mIL-6R (membrane receptor) blockage with tocilizumab used in COVID-19-related cytokine release syndrome may reduce the mortality [3]. This has been confirmed by several retrospective cohort studies and case series from China and Europe [4–10]. The Polish Association of Epidemiologists and Infectiologists recommendations include TCZ as an option for COVID-19 treatment [11]. Therefore, the Hospital for Infectious Diseases in Warsaw established a Standard Operating Procedure (SOP) for its use in this specific indication. Patients were qualified for TCZ if having PCR-confirmed SARS-CoV-2 infection with evidence of COVID-19 pneumonia on chest X-ray, increased IL-6 level and peripheral oxygen saturation (SpO_2_) ≤ 93% or PAO_2_/FiO_2_ ratio < 200 mmHg. For each patient fulfilling inclusion criteria risk–benefit ratio was verified by a committee of three physicians including patient’s physician, intensive care specialist and SOP coordinating physician. This process included verification of any evidence of severe infection other than SARS-CoV-2, as well as review of exclusion criteria. The recommended dosing was 8 mg/kg with a maximum dose of 800 mg per infusion and a second dose of TZC allowed after 8–12 h. The SOP was approved by Bioethical Committee of Medical University of Warsaw (nr KB68/2020).

## Case presentation

A 27-year-old, otherwise healthy man with no health risks, was admitted to infectious disease ward with a week history of weakness, fever and sore throat. Due to these conditions he was treated with amoxicillin and clarithromycin with no clinical improvement. On admission the patient reported fever, cough and dyspnea. He had no concomitant chronic conditions and did not take any prescribed medication. On examination he was overweight (body mass 86 kg, BMI 27.8 kg/m^2^), body temperature of 39.9 °C degrees, blood pressure 138/83 mm Hg, HR 122/min. and respiratory rate 20/min. His peripheral oxygen saturation (SpO_2_) was 88% and improved to 95% on 6 l/min. oxygen supplementation.

**Fig. 1:**
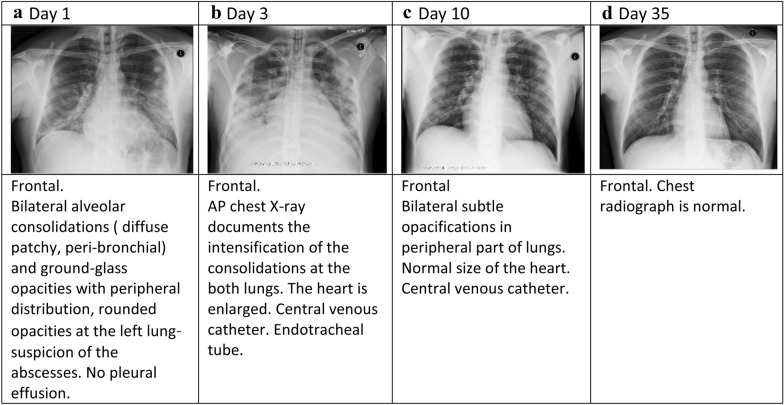
The evolution of pulmonary infiltrates in X-ray

**Fig. 2:**
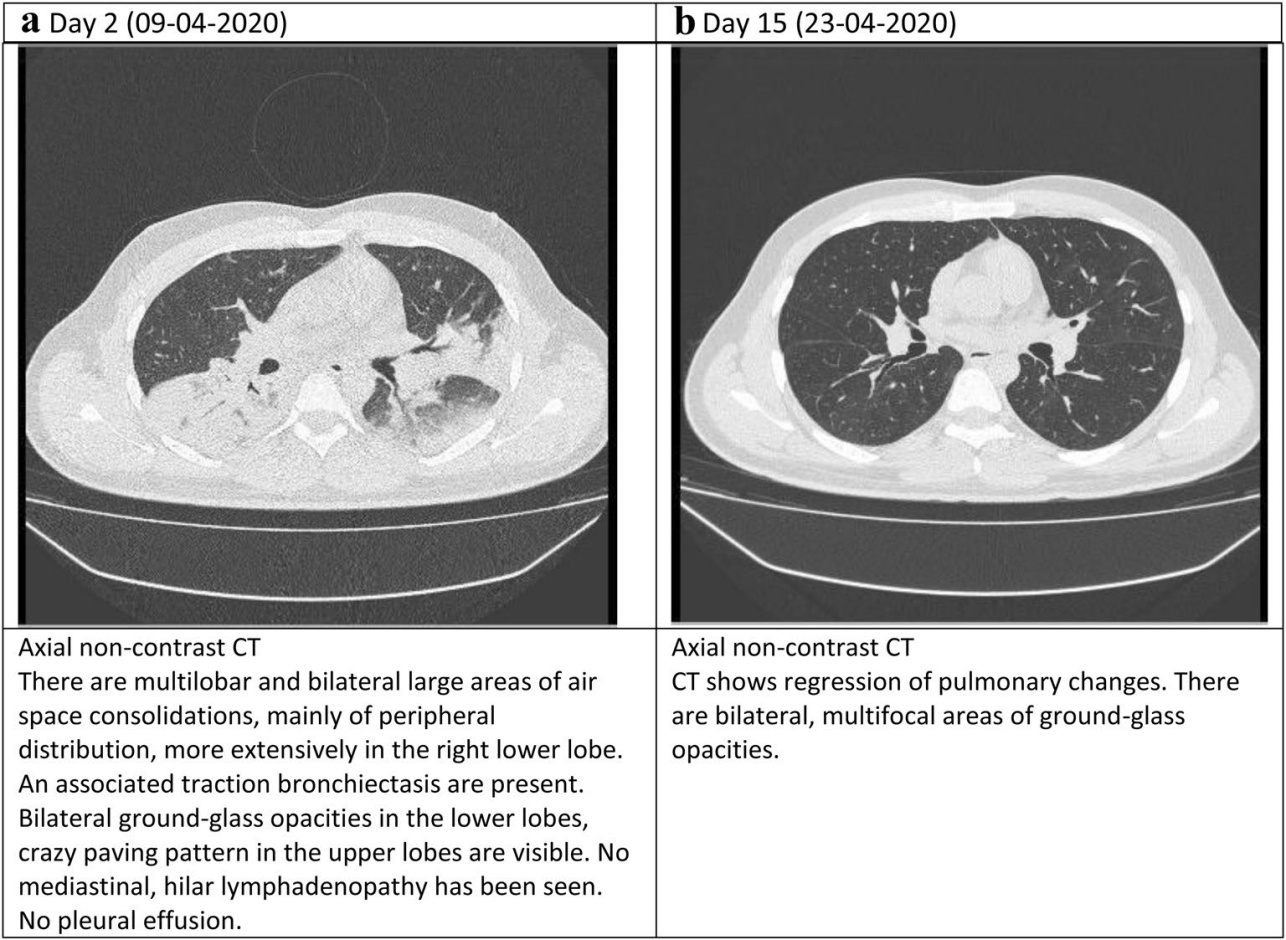
The evolution of pulmonary infiltrates in computer tomography

A nasal swab was obtained on admission and reverse transcriptase-polymerase chain reaction (RT-PCR) assay confirmed SARS-CoV-2 infection. On baseline the patient was anti-HIV, anti-HCV and HBsAg negative. Monitoring of ferritin, C-reactive protein (CRP), fibrinogen, D-dimers and interleukin 6 (IL-6) was performed (Table 1). The patient received 500 mg of chloroquine twice daily and 500 mg of azithromycin once daily, according to earlier approved local standard of care. Due to initial suspicion of the lung abscess formation seen on the first chest X-ray, vancomycin was added (Fig. 1a). His computer tomography revealed bilateral changes typical for COVID-19 pneumonia and did not confirm changes characteristic for bacterial infection (Fig. 2a). In addition, low molecular weight heparin in prophylactic doses was started.

**Table 1:** Body weight and laboratory values measured over time in reported patient

	Day 1 On admission	Day 2 Before 1st TCZ dose	Day 3 Before 2nd TCZ dose	Day 3 After 2nd TCZ dose	Day 5	Day 7	Day 9	Day 12	Day 16	Day 35
Body weight (kg)	86	–	–	–	–	–	–	–	77	79
SARS-CoV-2 RT-PCR^a^ nasopharyngeal swab	Positive	–	–	–	–	Negative	Negative	–	–	Negative
SARS-CoV-2 antibodies^b^ (AU/ml)	–	–	–	–	–	IgM 3.89 IgG 129	–	–	–	IgM 2.05 IgG 63.77
IL-6 (pg/ml)	177	298.9	1836	1479	–	98.2	86.2	42	28.3	37.1
CRP (mg/l)	262	374	363	251	67	19	13	7	< 5	< 5
Ferritin (ng/ml)	–	1615	5076.3	–	–	1454.3	1680.5	860.9	–	199.4
D-dimers (ng/ml)	2007.15	2208.97	2336.06	2363.43	2821.55	2440.13	1587.5	564.19	249.43	110.14
Fibrinogen (g/l)	8.26		7.37			4.54		4.15		
Procalcitonin (ng/ml)	1.12	1.00	1.56	1.39	1.12	< 0.05	< 0.05	< 0.05	< 0.05	< 0.05
Leukocyte count (× 10^3^/mm^3^)	4.3	5.7	3.9	3.7	3.3	5.4	6.1	5.4	3.7	4.6
Neutrophile count (× 10^3^/mm^3^)	3.76	3.35	3.03	2.71	1.83	3.38	3.99	2.57	1.24	1.94
Platelet count (× 10^3^/mm^3^)	127	146	169	162	317	429	502	479	320	178
Hemoglobin (g/dl)	13.7	12.1	11.8	12.2	13.6	13.5	12.8	13.4	13.2	12.9
ALT (U/l)	74	61	–	50	–	304	236	209	180	124
AST (U/l)	91	74	–	61	–	168	125	74	45	45
GGTP (U/l)	137	165	–	141	–	547	514	336	214	96
Serum creatinine (mmol/l)	83	81	53	42	110.9	134	125	81	114	86

^a^ SARS-CoV-2 Genesing Real-Time PCR Assay. Test Vitassay ^b^ quantitative chemiluminescence test (CLIA) on Maglumi analyzer by SNIBE (China). Distributed by CORMAY S.A

Within several hours from admission and irrespective of oxygen supplementation of 15 l/min, his SpO_2_ dropped to 90%. He was diagnosed with ARDS, intubated and admitted to intensive care unit. There he was qualified to receive TCZ according to SOP (two doses of 800 mg). The patient was ventilated with P/SIMV (FiO_2_ 1,0-0,9-1,0, f 20, Pi20, PEEP 12) and received standard empiric antibiotic therapy (meropenem and continuation of vancomycin). Patient’s state did not change substantially over the next 4 days, however his body temperature normalized and CRP dropped. At the same time, his pulmonary infiltrates intensified on the control X-ray (Fig. 1b). However, his SpO_2_ steadily improved as presented in Table 1. After TCZ infusion IL-6 levels increased over ten times, but decreased within the next 3 days (Table 1). In terms of adverse effects the patient had transaminases and GGTP flair, as well as kidney function deterioration on day 7, i.e., 4 days after TCZ infusion (Table 1). On the fifth day the patient’s state improved significantly and he was extubated. After moving back to infectious disease department, the patient continued to improve with normalization of inflammatory markers. The patient was discharged from the hospital on day 21 after confirming significant improvement in his CT scan and two negative SARS-CoV-2 RT-PCR from nasopharyngeal swabs (Fig. 2, Table 1).

A follow-up hospitalization on day 35 revealed no radiological changes on chest X-ray, negative SASR-CoV-2 RT-PCR from nasopharyngeal swab. However, the level of IL-6 and alanine aminotransferase activity were increased. The patient reported improving tolerance for physical activity, but he was unable to perform his previous activities with the same strength, e.g., singing.

## Discussion and conclusions

Data on COVID-19 treatment effectiveness and long-term safety are currently sparse. Post-hospitalization follow-up is neither recommended nor their outcomes available in the current publications. Most work in the post-hospitalization area focuses on epidemiological considerations and psychological well-being [12]. In such events, case studies remain an important area of knowledge sharing and could support building expertise in the field where evidence-based approach is not yet available. Here, we present a case of severe COVID-19 in a 27-year-old, otherwise healthy patient. Although we observed full recovery in clinical, radiological and viral terms, some important adverse effects were present during hospitalization and afterwards. In terms of kidney and hepatic injury in the presented patient, it is uncertain whether this was an effect of prescribed treatment (chloroquine, antibiotics and TCZ), hyperinflammation or post-hypoxic organ injury [13–15].

Guaraldi et al. showed that TCZ treatment was associated with a 40% reduction in the risk of invasive mechanical ventilation or death, but at the same time 13% of patients treated with TCZ were diagnosed with new infections, as compared to only 4% of patients treated with standard of care alone [16]. It clearly shows that the risk–benefit ratio for this approach is uncertain and needs to be further evaluated by randomized controlled trials, which are currently ongoing [17, 18]. In our patient, elevated IL-6 was observed even on day 35, which suggests a residual IL-R6 receptor blockage. As TCZ is anti-inflammatory agent a delay in the diagnosis of infection due to reduced fever and inflammation markers is likely [19]. These overlapping factors warrants careful long-term follow-up of COVID-19 patients in terms of adverse effects of medical interventions.

As reported by Phua et al. in a systematic review, 30% of patients survive ARDS and 70% of survivors will live with significant disability [20]. It remains uncertain to what extent this applies to COVID-19 survivors. Although our case report shows that young patients with no concomitant diseases can present full ‘radiological’ recovery, we were not able to perform any functional lung tests, such as spirometry or plethysmography, due to epidemiological restrictions. Data on a longer term pulmonological assessment and follow-up of COVID-19 survivors are sparse. In addition, it remains on research peripheries and outside recommendation area. Therefore, we introduced to our local practice two planned follow-up visits: 6 weeks after discharge from the hospital to verify immediate complications and after another 6 weeks to check on patients full recovery or any disabilities.

Another area lacking precision in most guidelines is when to introduce anti-inflammatory treatment and how to monitor its effectiveness and safety. Only few studies investigated an IL-6 level in which TCZ could be indicated in COVID-19 context. Herold et al. proposed a range of 40–80 pg/ml, but most clinical trials do not provide IL-6 threshold guided treatment and follow-up [17, 21].

As for the effectiveness and safety although ferritin, CRP, fibrinogen and D-dimers levels may be indicative of hyperinflammation these markers are highly nonspecific. IL-6 monitoring seems to be more accurate as a marker of immune system activation, but its levels are highly dependent on age, concomitant diseases (e.g., cancers) and host genetics [22]. Therefore, all these markers need to be monitored frequently to capture the dynamic and predict incoming cytokine storm, but should not mitigate the clinical symptoms progression. As presented also in our case report, IL-6 levels indicate the effectiveness of receptor blockage after TCZ infusion. They have been also proposed as a good prognostic marker after TCZ infusion [6, 7]. Herold et al. presented maximal level of IL-6 followed by CRP level as highly predictive of the need for mechanical ventilation [21]. Therefore, in our experience, all inflammatory markers in symptomatic COVID-19 patients should be monitored at least daily. A decision on TCZ treatment should be made by a multidisciplinary group and individualized for a given patient taking into account the evolution of clinical presentation, dynamic in inflammatory markers and the risk related to concomitant diseases or infections. Currently most interventions are used in COVID-19 experimentally and/or “off label”, therefore careful risk to benefit assessment needs to be performed for each patient individually in order to ascertain minimum harm with maximal benefit.

A standardized approach to follow-up for COVID-19 survivors is urgently needed. Current and future research should also investigate the impact of experimental therapies on lung tissue healing and regeneration, as well as long-term treatment toxicities.

## Data Availability

Not applicable.
